# Publisher's Note: Addendum to Volume 26, Issue Supplement 1, December 2025, International Conference on Genome Informatics ISCB-Asia 2025 Abstract Book

**DOI:** 10.1093/bib/bbag026

**Published:** 2026-03-10

Addendum to Volume 26, Issue Supplement 1, December 2025, International Conference on Genome Informatics ISCB-Asia 2025 Abstract Book (https://academic.oup.com/bib/issue/26/Supplement_1)

Due to an error in the conference submission system, four abstracts were omitted from the supplement and instead have been published in this issue of the journal. The abstracts are published at:

https://doi.org/10.1093/bib/bbag055

https://doi.org/10.1093/bib/bbag056

https://doi.org/10.1093/bib/bbag057

https://doi.org/10.1093/bib/bbag058

